# CD4 T-cell aging exacerbates neuroinflammation in a late-onset mouse model of amyotrophic lateral sclerosis

**DOI:** 10.1186/s12974-023-03007-1

**Published:** 2024-01-11

**Authors:** Shir Zaccai, Anna Nemirovsky, Livnat Lerner, Leenor Alfahel, Ekaterina Eremenko, Adrian Israelson, Alon Monsonego

**Affiliations:** 1 Department of Physiology and Cell Biology, Faculty of Health Sciences and The School of Brain Sciences and Cognition, Ben-Gurion University of the Negev, P.O.B. 653, 84105 Beer Sheva, Israel; 2 The Shraga Segal Dept. of Microbiology, Immunology and Genetics, Faculty of Health Sciences and The School of Brain Sciences and Cognition, Ben-Gurion University of the Negev, P.O.B. 653, 84105 Beer Sheva, Israel

**Keywords:** Amyotrophic lateral sclerosis, SOD1 mice, Neuroinflammation, CD4 T cells, Microglia, Antigen presentation

## Abstract

Amyotrophic lateral sclerosis (ALS) is an adult-onset progressive neurodegenerative disorder characterized by the loss of upper and lower motor neurons in the brain and spinal cord. Accumulating evidence suggests that ALS is not solely a neuronal cell- or brain tissue-autonomous disease and that neuroinflammation plays a key role in disease progression. Furthermore, whereas both CD4 and CD8 T cells were observed in spinal cords of ALS patients and in mouse models of the disease, their role in the neuroinflammatory process, especially considering their functional changes with age, is not fully explored. In this study, we revealed the structure of the CD4 T-cell compartment during disease progression of early-onset SOD1^G93A^ and late-onset SOD1^G37R^ mouse models of ALS. We show age-related changes in the CD4 T-cell subset organization between these mutant SOD1 mouse models towards increased frequency of effector T cells in spleens of SOD1^G37R^ mice and robust infiltration of CD4 T cells expressing activation markers and the checkpoint molecule PD1 into the spinal cord. The frequency of infiltrating CD4 T cells correlated with the frequency of infiltrating CD8 T cells which displayed a more exhausted phenotype. Moreover, RNA-Seq and immunohistochemistry analyses of spinal cords from SOD1^G37R^ mice with early clinical symptoms demonstrated immunological trajectories reminiscent of a neurotoxic inflammatory response which involved proinflammatory T cells and antigen presentation related pathways. Overall, our findings suggest that age-related changes of the CD4 T cell landscape is indicative of a chronic inflammatory response, which aggravates the disease process and can be therapeutically targeted.

**Supplementary Information:**

The online version contains supplementary material available at 10.1186/s12974-023-03007-1.

## Background

ALS is a fatal neurodegenerative disease characterized by the progressive loss of upper and lower motor neurons in the brain and spinal cord (SC) [1]. A number of risk factors for ALS have been hypothesized; however, the only recognized risk factors to date are older age, male sex, and a family history [2]. About 90% of ALS cases are sporadic, occurring without a known genetic cause. However, the remaining 10% are familial cases that are usually inherited in a dominant manner [1]. The *SOD1* gene, which encodes for Cu/Zn superoxide dismutase, was the first gene found to be associated with ALS [3]. Mutations in this gene are responsible for approximately 20% of familial ALS cases and about 2% of sporadic ALS cases, making *SOD1* one of the most studied genes in this disease [4, 5]. Different mutant SOD1 transgenic mice develop a disease with a similar pathogenesis to ALS, which includes motor neuron degeneration, neuroinflammation, paralysis and early death [4].

The pathology of ALS is not limited to neuronal cell-autonomous mechanisms [6]. For example, it has been shown that mice overexpressing human mutant SOD1 protein only in neurons, did not develop motor impairment [7] or had a late disease onset [8]. Further experiments showed that specific deletion of mutant SOD1 from motor neurons delayed disease onset and early phase of disease progression with no effect on the late phase of the disease [9, 10]. In contrast, deletion of mutant SOD1 from astrocytes or microglia strongly delayed the late phase of the disease [9, 11]. Therefore, non-neuronal cells play an important role in motor neuron loss and disease progression [10, 12].

Neuroinflammation is an essential host defense mechanism to protect the brain from infection or injury and restore normal structure and function [13, 14]. However, chronic inflammation can induce cytotoxicity and worsen severity of different neurodegenerative diseases such as Parkinson’s disease (PD) [15], Alzheimer’s disease (AD) [16], multiple sclerosis (MS) [17] and ALS [14, 18]. Neuroinflammation is characterized by increased microglial and astrocytic activation, leukocyte infiltration into the brain and SC and elevated levels of proinflammatory cytokines [18, 19]. In the context of ALS, neuroinflammation plays a critical role in the disease progression [18]. The dysregulated inflammatory responses, characterized by aberrant microglial activation and excessive proinflammatory cytokines, contribute to the neurodegeneration observed in ALS [20].

Microglia, as the resident macrophages of the central nervous system (CNS), play a pivotal role in maintaining brain homeostasis [21]. However, under pathological conditions, these cells can transition from their surveillant homeostatic state to various activation states including subsets which exhibit antigen presenting cell (APC) phenotypes [22, 23]. Through their MHCII-based interactions with various CD4 T-cell subsets (e.g., proinflammatory Th1 or Th17 cells [24] or anti-inflammatory Tregs [25]), microglia can contribute to inflammation-driven neuronal damage or repair. However, in the context of ALS, the nature and consequences of such interactions remain largely unknown.

In mutant SOD1^G93A^ mice, CD4 T helper cells were observed in lumbar SC at early disease stage, whereas at end stage, both CD4 and CD8 T lymphocytes were present [18, 26, 27]. Genetic deletion of functional T cells or CD4 T cells resulted in accelerated disease progression, increased mRNA levels of NOX2 (NADPH oxidase isoform which catalyze the production of superoxide molecules) and proinflammatory cytokines, and decreased levels of the neurotrophic factors IGF-1, BDNF and GDNF [27]. Reconstitution of T cells by bone marrow transplantation, prolonged the survival, reduced proinflammatory responses, restored neuroprotective factors and inhibited M1 microglial activation [18, 27]. In ALS patients, peripheral blood cell counts of CD3, CD4, CD8 and CD3^+^CD56^+^ T cells, natural killer cells and granulocytes were increased [28]. Moreover, whereas a higher frequency of effector CD4 T cells in both blood and CSF was linked to decreased survival, increased frequencies of activated regulatory T (Treg) cells in the blood was associated with improved survival [29].

While the SOD1^G93A^ mouse model has been widely used in ALS research primarily due to rapid progression of symptoms [30], such early onset of disease may not represent the impact of age-related immunological changes as they appear in humans. The CD4 T-cell subsets are critical mediators of immune responses, and their phenotypic and functional diversity have been shown to be modulated by age [31–33].

In this research, we hypothesized that there is a significant age-related CD4 T-cell component in ALS which impacts the neuroinflammatory response in the CNS. We observed distinct organization of CD4 T-cell subsets between early-onset and late-onset SOD1 mouse models. Moreover, our findings establish a positive correlation between effector memory (EM) and cytotoxic CD4 T cells with disease progression. In addition, we show upregulation in expression of genes and proteins involved in formation of immunological synapse. Taken together, these findings emphasize the significant influence of effector CD4 populations on the progression of the disease in late-onset mutant SOD1^G37R^ mice.

## Methods

### Mice

Transgenic mice expressing human SOD1^G93A^ [30] and loxSOD1^G37R^ [9] were bred with C57BL6 mice and genotyped by PCR of DNA extracted from tails. All mice were maintained in the animal facility of Ben-Gurion University of the Negev using standard protocols. All procedures involving animals were consistent with the requirements of the Animal Care and Use Committees of Ben-Gurion University of the Negev.

### Tissue collection and processing

*Spleen tissue collection.* SOD1^G93A^ and SOD1^G37R^ mice were killed using overdose of isoflurane. Immediately after, spleens were harvested, and blood was collected following incision of the mouse right atrium. Next, perfusion was performed using 20 ml of ice-cold Dulbecco’s phosphate buffered saline (PBS) and SC was collected. Both spleens and SCs were weighed and kept in HBSS solution on ice.

*Tissue processing—spleens.* Spleens were mashed into a 70-µm cell strainer and 300 µl of ammonium-chloride-potassium (ACK) buffer (Lonza, Switzerland) was added for 1.5 min to lyse red blood cells. For flow cytometry and splenocyte culture, cells were diluted into 2,500,000 cell/ml in RPMI 1640 media (Thermo Fisher Scientific, MA, United States), supplemented with of 10% fetal bovine serum (FBS) (Thermo Fisher Scientific, MA, United States), HEPES buffer solution (10 mM) (Sartorius, Germany), 1 mM sodium pyruvate solution (Sartorius, Germany), 10 mM MEM non-essential amino acids solution (Sartorius, Germany), 1% penicillin–streptomycin solution (Sartorius, Germany), and 50 μM 2-mercaptoethanol, Molecular Biology Grade (Sigma-Aldrich, MO, United States).

*Spinal cord tissue collection.* SOD1^G93A^ and SOD1^G37R^ mice were killed using overdose of isoflurane perfused with cold PBS. Immediately after, SCs were collected by hydraulic extrusion. The procedure involved isolating the spinal column by decapitating the head and cutting proximal to the pelvic bone. Next, a syringe filled with PBS was carefully positioned at the distal end of the spinal column and steady pressure was applied to the syringe facilitating the extrusion of the SC.

*Tissue processing—spinal cord.* Upon extrusion of the SCs, they were placed in Hanks' Balanced Salt Solution (HBSS) (Sartorius, Germany) and kept on ice. Then, to dissociate the tissue, the Neural Tissue Dissociation Kit (Miltenyi Biotec, Germany) was used following the manufacturer’s instructions, with slight adjustments to the protocol. Specifically, we excluded the papain incubation step, as it was found to impact the integrity of specific cell surface epitopes. Instead, to ensure tissue dissociation, we added physical dissociation step using a glass pipette, in combination with passing the tissue through a 70-μm cell strainer to eliminate larger particles.

### Flow cytometry

*For extracellular staining*, cells were washed 3 times with FACS Buffer (2% fetal bovine serum and 1 mM EDTA in PBS). Then, cells were incubated with TruStain FcX (Biolegend, CA, United States) for 5 min in 4°C. Next, cells were incubated with antibody mix for 20 min in 4 °C. *For SC* cells, the antibodies that were used for extracellular membranal antigen staining are detailed in Additional file 2: Table S1. After staining with extracellular antibodies, cells were washed 3 times and were run using CytoFLEX LX instrument (Beckman Coulter, CA, United States).

*For splenocytes*, the antibodies that were used for extracellular membranal antigen staining are detailed in Additional file 2: Table S1. After staining with extracellular antibodies, splenocytes were washed 3 times, fixed and permeabilized using Foxp3/Transcription Factor Staining Buffer Set (Thermo Fisher Scientific, MA, United States) following manufacturer’s instructions. The next day, cells were washed 3 times with permeabilization buffer and blocked using rat serum (2 µl for 90 µl permeabilization buffer) for 5 min in room temperature. Then, cells were incubated with intracellular antibody mix for 20 min at room temperature. The antibodies that were used for intracellular staining are detailed in Additional file 2: Table S1. Next, cells were washed 3 times with permeabilization buffer and FACS staining buffer was added in the last wash and samples were run using CytoFLEX LX instrument (Beckman Coulter, CA, United States). Data were analyzed using CytExpert Acquisition and Analysis Software 2.4 (Beckman Coulter, CA, United States) and FlowJo software v10.7 (BD, NJ, United States). Gating strategies excluded dead cells, clumps, and debris and were set based on fluorescence minus one (FMO) and unstained samples for splenocytes and unstained samples for SC cells. All CD4 T-cell populations were gated from lymphocytes (SSC-A vs FSC-A), live cells (Fixable Viability Dye vs FSC-A), single cells (SSC-A vs SSC-H) and were calculated as percentage from CD3^+^CD4^+^ or from effector memory (EM) (CD44^+^CD62L^−^) populations (Additional file 1: Fig. S1A).

### Bulk RNA sequencing

Mice were euthanized with an overdose of isoflurane and perfused with cold PBS. Then, SCs were extracted as detailed in tissue collection and processing section, transferred into RNase-free tubes and immediately cryopreserved using liquid nitrogen. RNA was extracted from lumbar region of SC using RNeasy plus mini kit (Qiagen, Germany), according to manufacturer’s instructions. RNA was quantified using Qubit (Thermo Fisher Scientific, MA, United States) and its integrity was assessed using Bioanalyzer (Agilent Technologies, CA, United States). RNA was diluted to a concentration of 5–10 ng/µl and MARS-Seq protocol was used to generate RNA-seq libraries [34, 35].

### RNA-Seq data analysis

Analysis was performed on combined data from 2 independent experiments with overall 10 SOD1^G37R^ mice and 10 littermate control mice. One experiment included 3 female SOD1^G37R^ mice, 3 female littermate control mice, 3 male SOD1^G37R^ mice and 3 male littermate control mice. The other experiment included 4 male SOD1^G37R^ mice and 4 male littermate control mice.

Initial analysis of the raw sequence reads was carried out using the NeatSeq-Flow platform [36]. The sequences were quality trimmed and filtered using Trim Galore (v0.4.5) and cutadapt (v1.15). Alignment of the reads to the mouse genome (GRCm38) was done using STAR (v2.5.2a) [37]. The number of reads per gene per sample was counted using RSEM (v1.2.28) [38]. Quality assessment (QA) of the process was carried out using FASTQC (v0.11.8) and MultiQC (v1.0.dev0) [39]. After trimming, each sample had on average 79M reads with an average sequence length of 74.4 bp.

Statistical testing for identification of differentially expressed genes, batch correction, gene annotation, clustering and enrichment analysis were performed with the DESeq2 module within the NeatSeq-Flow platform [36]. Batch correction was done using the SVA/Combat R package. Gene annotation was done using the "AnnotationHub" R package (snapshot: 2020–04-27). The statistical analysis was performed using the DESeq2 [40] R package. For comparison (Contrast) between groups, the statistical model considered two effects: the treatment group and the batch (experiment). The analysis produced *P*-adjusted, and fold of change (FC) per gene. Genes with *P*-adjusted < 0.05 were considered Differentially Expressed (DE). KEGG Enrichment analysis was performed using the clusterProfiler [41] (v3.16.0) R package.

### Immunohistochemistry

Mice were euthanized with an overdose of isoflurane and perfused with cold PBS. Then, SCs were extracted as detailed in tissue collection and processing, immersed in 4% paraformaldehyde solution at 4 °C overnight, transferred to a freshly prepared 30% sucrose solution at 4 °C for 24 h and fixed in OCT (Tissue-Tek, Torrance, CA). Transverse sections (40 µm) of SCs were produced using a Leica CM3050S Research Cryostat (Leica Biosystems, Germany) with object and chamber temperature of − 25 °C. Sections kept at − 20 °C in cryopreservation solution (25% glycerol, 25% ethylene glycol, and 50% PBS). Sections were rinsed twice in 0.05% Tween 20 washing solution and permeabilized in a 0.5% Triton X-100 solution for 30 min. Prior to staining, sections were blocked with 10% serum of secondary antibody and 1% BSA solution. The primary antibodies that were used for immunohistochemistry staining are detailed in Additional file 3: Table S2. The antibodies used for secondary staining are detailed in Additional file 3: Table S2.

### Confocal image analysis

Confocal images were generated with a 4-channel Olympus XI81-ZDC confocal microscope (Olympus, Hamburg, Germany) at a 1024 × 1024pixel resolution with ×20, or ×60 objectives. To generate 3D images, z-stack of at least 40 μm thickness, with serial images taken every 1 μm, was imaged using confocal microscope and reconstructed using IMARIS 9.8 software (Oxford Instruments, United Kingdom).

### Statistical analysis

Data are presented as mean ± SEM. Statistical evaluations were performed using the non-parametric Mann–Whitney U test, Spearman's rank correlation test, and Wilcoxon matched-pairs signed rank test  Further details of the RNA-Seq statistical analysis are detailed in RNA-Seq data analysis section. All analyses were executed with a *P* < 0.05 threshold for statistical significance.

## Results

### CD4 T-cell subsets in spleens of symptomatic late-onset SOD1^G37R^ mice exhibit enhanced effector functions compared with symptomatic early-onset SOD1^G93A^ mice

**Fig. 1:**
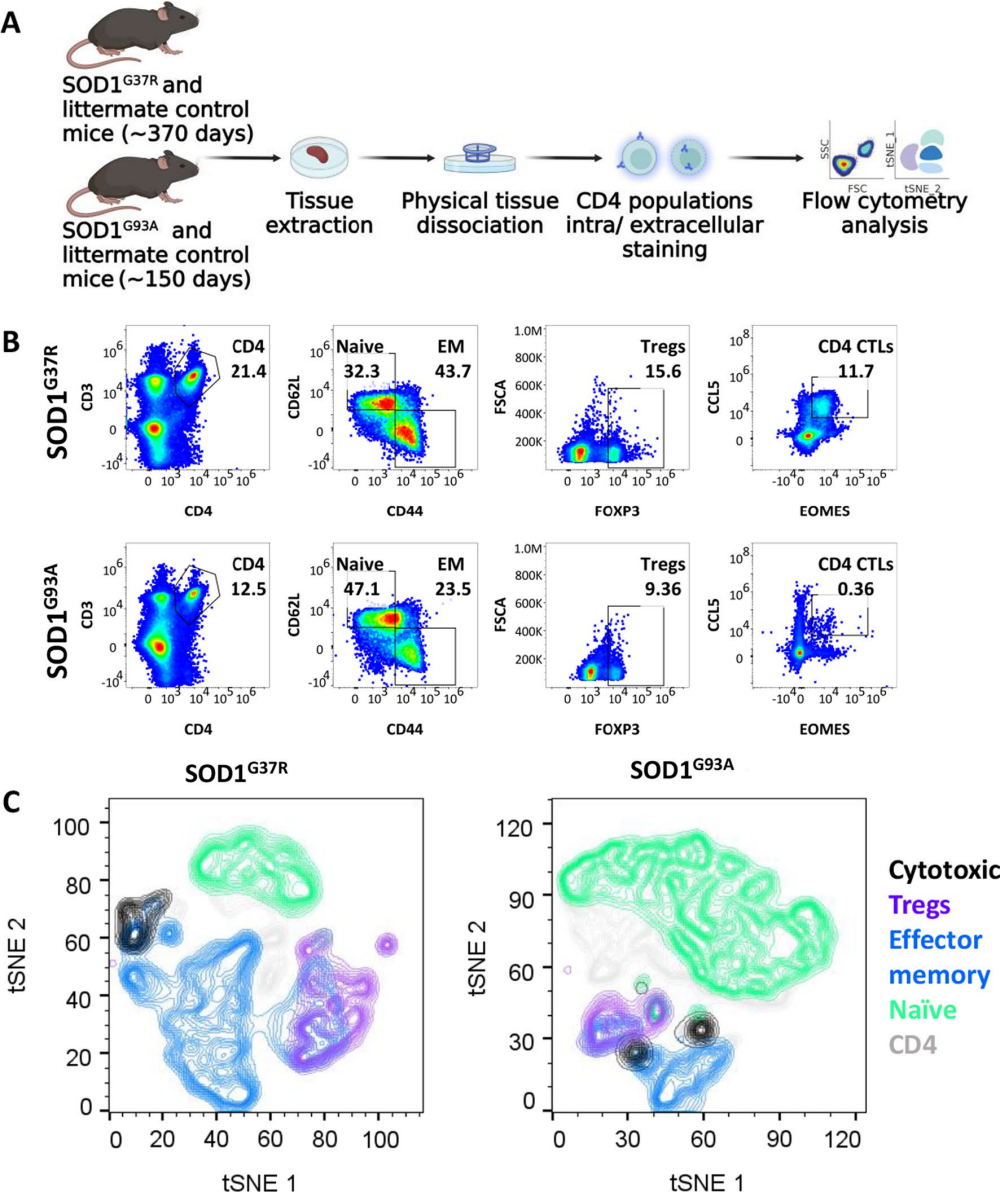

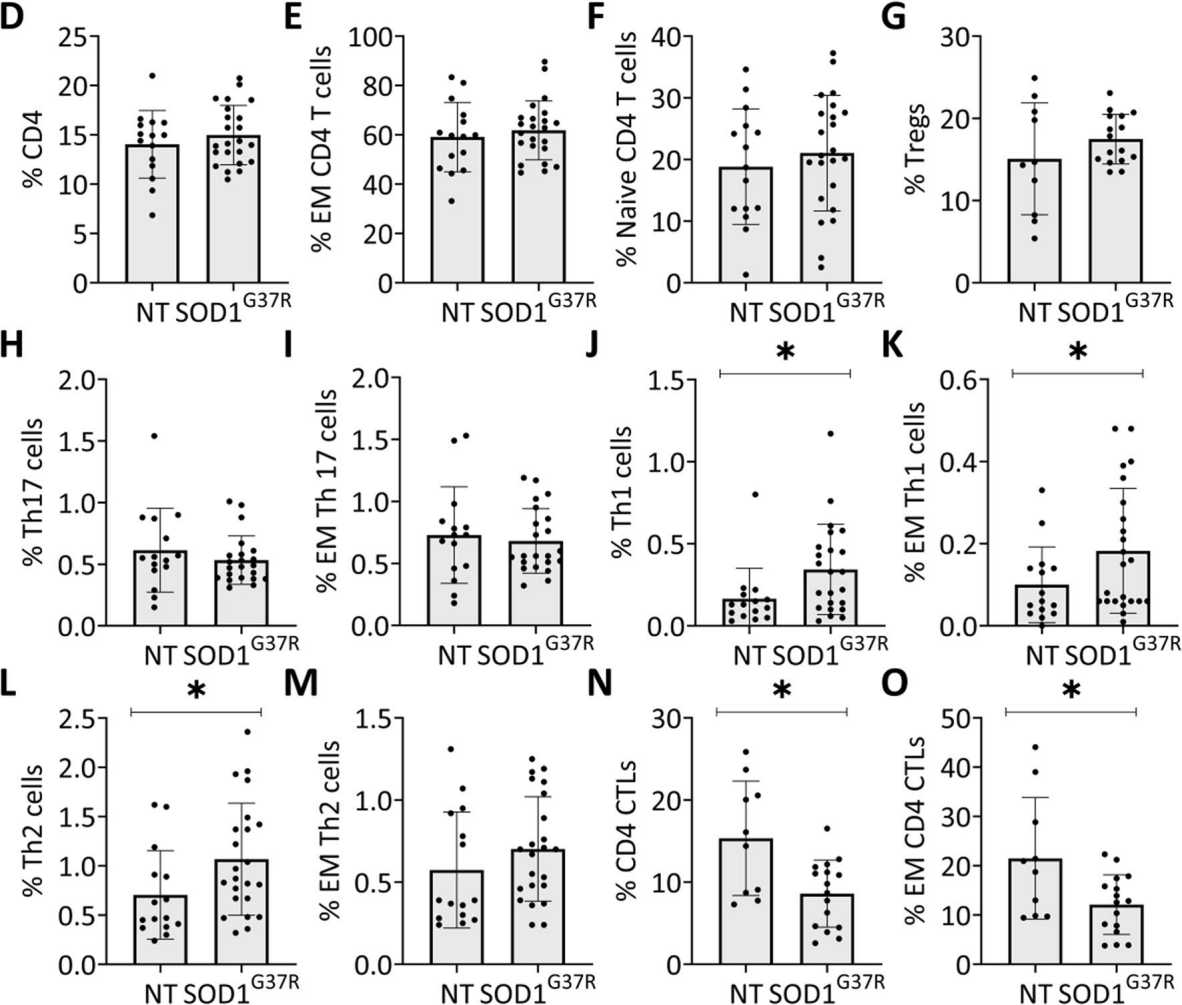
Increased frequencies of effector CD4 T-cell subsets in spleens of symptomatic, late-onset, SOD1^G37R^ mice. **A** Splenocytes from mutant SOD1^G37R^ and SOD1^G93A^ mice were mechanically dissociated, immunostained for CD4 T-cell markers, and analyzed with flow cytometry. CD4 T-cell populations were gated from lymphocytes (SSC-A vs FSC-A), live cells (Fixable Viability Dye vs FSC-A), and single cells (SSC-A vs SSC-H). **B** CD4 T-cell subsets in late-onset SOD1^G37R^ (upper panels) and early-onset SOD1^G93A^ (lower panels) mice (gating strategy is shown in Additional file 1: Fig. S1A). **C** tSNE plot of CD4 T-cell subset distribution in symptomatic late-onset SOD1^G37R^ (*n* = 3) and in early-onset SOD1^G93A^ mice (*n* = 3). **D**–**O** CD4 T-cell subset frequency in spleens of NT mice (*n* = 15, **D**–**F** and **H**–**M**; *n* = 10, **G**, **N** and **O**) and SOD1^G37R^ mice (*n* = 23, **D**–**F** and **H**–**M**; *n* = 16, **G**, **N** and **O**), shown as percentage of CD4 or EM CD4 T cells (mean ± SEM). *P ≤ 0.05, two-tailed Mann–Whitney U-test

Splenocytes were isolated from spleens of symptomatic SOD1^G37R^ mice (stage 2, approximately 370 days of age) and SOD1^G93A^ (stage 2, approximately 130 days of age), during which foot-dragging or toe-curling is observed during walking [42] (Fig. 1A).

To assess general differences in CD4 T-cell distribution between late-onset SOD1^G37R^ and early-onset SOD1^G93A^ mice, tSNE analysis of flow cytometry data was performed. tSNE plot indicates differences in CD4 T-cell subset distribution between both models **(**Fig. 1C**)**. Compared with early-onset SOD1^G93A^ mice, splenocytes from late-onset SOD1^G37R^ mice exhibited increased frequencies of EM CD4 T cells, Tregs and cytotoxic CD4 T cells (CTLs) along with reduced frequencies of the naïve population (Fig. 1C and Additional file 4: Table S3), similar to littermate controls (Additional file 1: Fig. S1B and Additional file 4: Table S3). When comparing to littermate controls, late-onset SOD1^G37R^ mice exhibited no difference in frequencies of CD4 (CD3^+^CD4^+^), EM (CD3^+^CD4^+^CD44^+^CD62L^−^), naïve (CD3^+^CD4^+^CD44^+^CD62L^−^), Tregs (CD3^+^CD4^+^FOXP3^+^), Th17 (CD3^+^CD4^+^RORγ^+^), and EM Th17 (CD3^+^CD4^+^CD44^+^CD62L^−^RORγ^+^) cells (Fig. 1D–I). Notably, splenocytes from the late-onset SOD1^G37R^ mice exhibited increased frequencies of proinflammatory Th1 (CD3^+^CD4^+^Tbet^high^), EM Th1 (CD3^+^CD4^+^ CD44^+^CD62L^−^Tbet^high^), anti-inflammatory Th2 (CD3^+^CD4^+^ GATA3^high^), but not EM Th2 (CD3^+^CD4^+ +^CD44^+^CD62L^−^GATA3^high^) cells **(**Fig. 1J–M); differences which were not observed in SOD1^G93A^ mice **(**Additional file 1: Fig. S1C-N and Additional file 4: Table S3). Interestingly, we also observed reduced frequencies of CD4 CTLs (CD3^+^CD4^+^CCL5^+^EOMES^+^) and EM CD4 CTLs (CD3^+^CD4^+^CD44^+^CD62L^−^CCL5^+^EOMES^+^) (Fig. 1N–O), cells which were recently implicated in aging and ALS [29, 31]. Together, our results reveal a shift with age and disease towards effector phenotypes of CD4 T cells in symptomatic late-onset SOD1^G37R^ which may aggravate disease progression.

### Effector phenotypes of CD4 T cells correlate with disease severity in SOD1^G37R^ mice

**Fig. 2:**
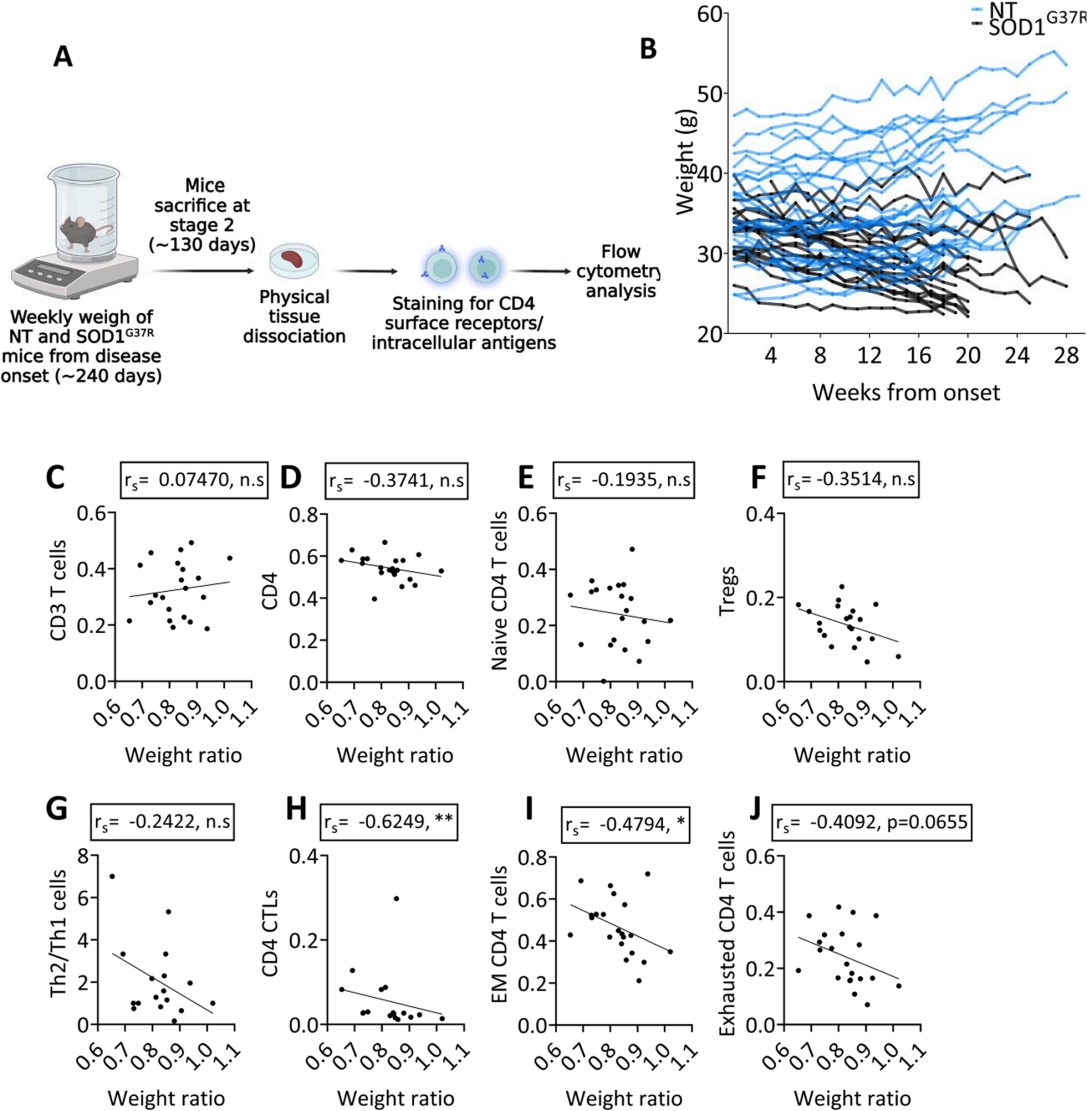
Cytotoxic and effector phenotypes of CD4 T-cell populations correlate with disease progression of mutant SOD1^G37R^ mice. **A** Mice were weighted from disease onset until symptomatic stage 2. Spleens were mechanically dissociated, immunostained for CD4 T-cell markers, and flow cytometry was performed. **B** Weight loss curves of SOD1^G37R^ mice starting from disease onset (approximately 240 days). **C**–**J** Correlations between CD4 T-cell populations and weight ratio in SOD1^G37R^ mice (*n* = 21, **C**–**G** and **I**–**J**; *n* = 17, **H**). *P ≤ 0.05, Spearman's rank correlation

To determine whether alterations in frequencies of CD4 T-cell subsets correlate with disease progression, SOD1^G37R^ mice were subjected to weekly weight assessment from disease onset. Upon reaching stage 2, mice were killed, and spleens were excised for flow cytometry analysis (Fig. 2A). As shown in Fig. 2B, weight plotted over time for both SOD1^G37R^ mice and age- and sex-matched littermate controls shows that individual SOD1^G37R^ mice variably lose weight during disease progression (Fig. 2B). To determine whether age- and disease-related CD4 T cell subsets contribute to disease severity, we correlated their frequency with weight ratio, defined as the weight at stage 2 (approximately 370 days old), divided by weight at 240 days old. Lower weight ratio values indicate enhanced disease progression in SOD1^G37R^ mice [42]. Notably, whereas the frequency of CD3+, CD4+, naïve CD4 T cells, Tregs, and Th2/Th1 ratio did not correlate with weight loss (Fig. 2C–G), frequencies of EM CD4 T cells and CD4 CTLs correlated with weight loss **(**Fig. 2H, I). In addition, there was a strong trend for the correlation between frequencies of exhausted CD4 T cells and weight loss **(**Fig. 2J). These findings highlight a potential role of circulating effector T cells and CD4 CTLs in shaping the disease course in SOD1^G37R^ mice.

### CD4 T cells infiltrate the spinal cord of SOD1^G37R^ mice exhibiting effector and exhausted phenotypes

**Fig. 3:**
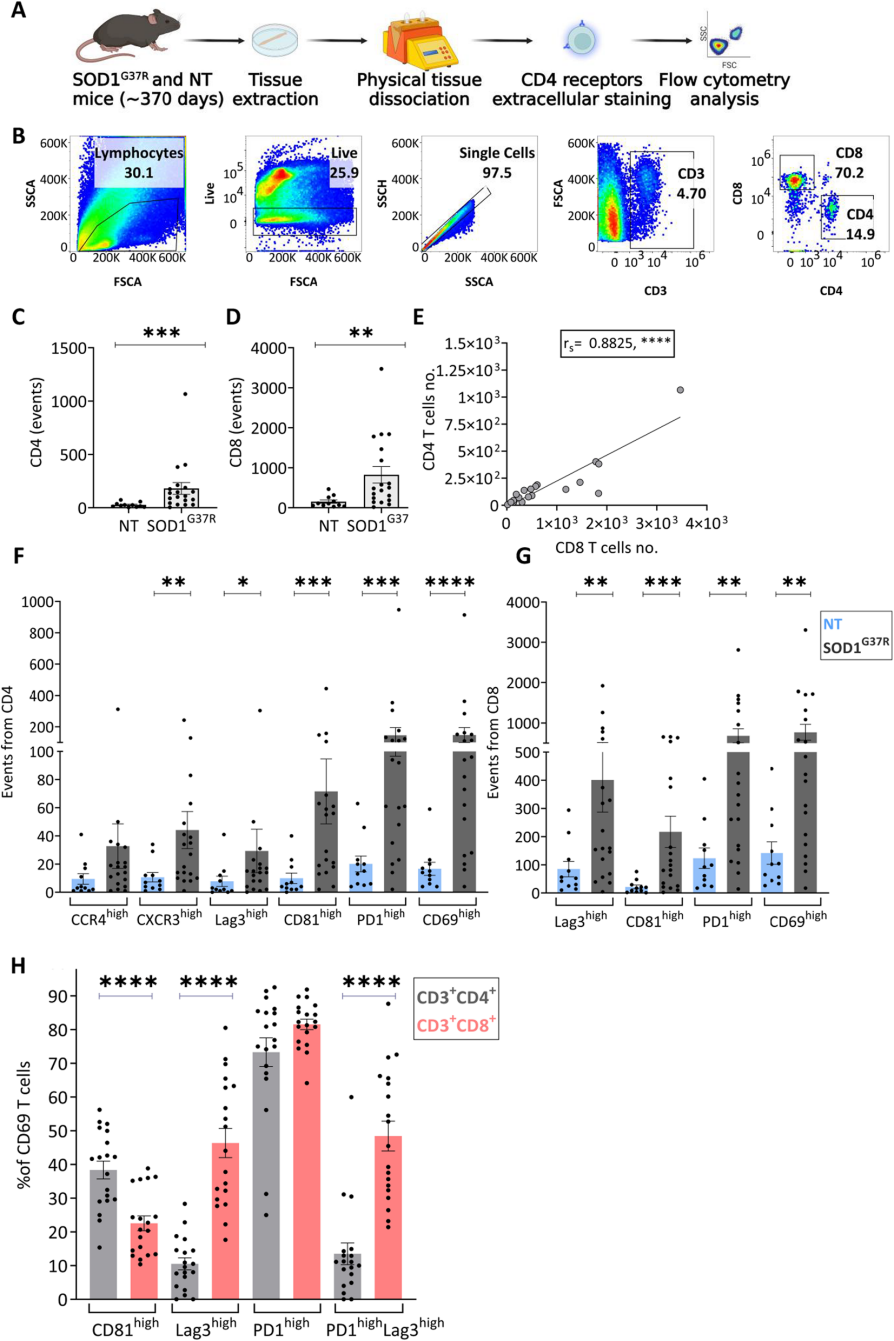
Activated and exhausted CD4 T cells infiltrate the spinal cord of mutant SOD1^G37R^ mice. **A** SCs from symptomatic mutant SOD1^G37R^ mice were mechanically dissociated and immunostained for flow cytometry. **B** Gating strategy for flow cytometry analysis; all T-cell populations were gated from lymphocytes (SSC-A vs FSC-A), live cells (Fixable Viability Dye vs FSC-A), single cells (SSC-A vs SSC-H). **C**, **D** Event number of CD4 and CD8 T cells in the SC of NT (*n* = 11) and symptomatic SOD1^G37R^ (*n* = 19) mice. **E** Correlation of event number of CD4 T cells and CD8 T cells. ****P ≤ 0.0001, Spearman's rank correlation. **F** Event numbers representing cells expressing CD4 T-cell receptors in SCs of NT (*n* = 11) and SOD1^G37R^ (*n* = 19) mice. **G** Event numbers representing cells expressing CD8 T-cell receptors in SCs of NT (*n* = 11) and SOD1^G37R^ (*n* = 19) mice. *P ≤ 0.05, **P ≤ 0.01, ***P ≤ 0.001, ****P ≤ 0.0001, Mann–Whitney U test. **H** Percentage of CD69^high^CD4^+^ and CD69^high^CD8^+^ T cells expressing activation and exhaustion receptors (mean ± SEM) in SC of symptomatic SOD1^G37R^ mice. ****P ≤ 0.0001, Wilcoxon matched-pairs signed rank test.

To determine whether the correlation of effector CD4 T cells with disease severity parallel the inflammatory process in the SC, we characterized the infiltrating T cells with flow cytometry. To that end, whole SCs were isolated from symptomatic mutant SOD1^G37R^ and littermate controls (stage 2, approximately 370 days of age) and symptomatic mutant SOD1^G93A^ (stage 2, approximately 130 days of age) and age-matched littermate controls, mechanically dissociated, and stained for surface receptors. All CD4 T-cell subsets were calculated as event number or percentage from CD3+ CD4+ or CD3+ CD8+ T cells (Fig. 3A, B). Whereas the event number of CD4 and CD8 T cells in whole SCs was greater in SOD1^G37R^ mice as compared with littermate controls (Fig. 3C, D), there was only a trend increase in SCs of SOD1^G93A^ mice (Additional file 1: Fig. S2). Furthermore, frequencies of infiltrating CD4 and CD8 T cells were strongly correlated in SOD1^G37R^ mice **(**Fig. 3E). Within the CD4 T-cell population of SOD1^G37R^ mice, event number of CXCR3^+^CCR6^−^ (Th1), CD81 (induced in formation of immunologic synapse) [43], PD1 and LAG-3 (inhibitory receptors) [44] and CD69 (activation marker) [43] were all increased compared with littermate controls (Fig. 3F). Similarly, within CD8 T cells, Lag3, CD81, PD1 and CD69 were highly expressed compared with controls (Fig. 3G). Notably, whereas most of the activated CD4+ CD69+ T cells (77.77% ± 2.34% of the infiltrating CD4 T cells) in the SC of SOD1^G37R^ mice were CD81+ (38.37% ± 2.65%), most of the activated CD8 T cells (91.74% ± 1.05% of the infiltrating CD8 T cells) were Lag3+ (46.38% ± 4.33) and PD1+ Lag3+ (48.44% ± 4.44%) (Fig. 3H), indicating a pronounced T-cell response with greater exhaustion of the CD8 subset.

### Neuroinflammation and T cell-mediated pathways are upregulated in spinal cords of mutant SOD1^G37R^ mice

**Fig. 4:**
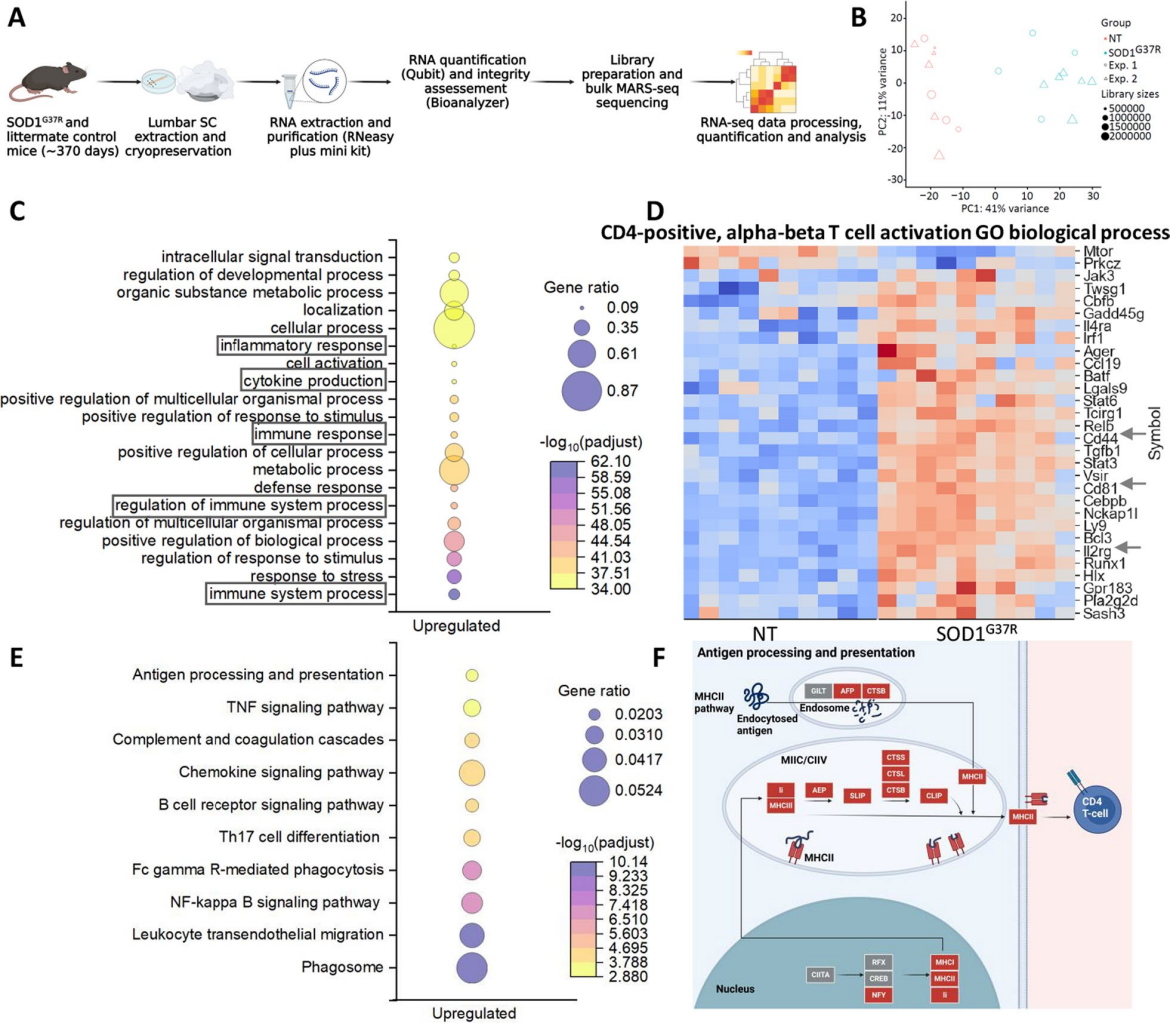
Immune and antigen presentation related pathways are upregulated in the spinal cord of mutant SOD1^G37R^ mice. **A** The lumbar region of the SC was extracted and cryopreserved, RNA was extracted and quantified, library was prepared and bulk RNA-seq was performed. **B** PCA analysis representing the gene expression profiles of symptomatic mutant SOD1^G37R^ mice (*n* = 10) compared with NT mice (*n* = 10). **C** Plot showing the ten most upregulated GO biological processes in the SC of symptomatic mutant SOD1^G37R^ mice compared with NT mice. **D** Heatmap of differentially expressed genes (see arrows) in the SC of symptomatic mutant SOD1^G37R^ and NT mice that are involved in CD4 T-cell activation biological processes (GO:0035710). **E** Upregulated immune-related KEGG pathways. **F** Genes upregulated (red) or unchanged (grey) in KEGG pathway for antigen processing and presentation

To explore the genes involved in disease progression of mutant SOD1^G37R^ mice, RNA was extracted from lumbar SCs of 10 SOD1^G37R^ and 10 littermate control mice and was subjected to bulk RNA-seq (Fig. 4A and Additional file 5: Table S4). A principal component analysis (PCA) was conducted and demonstrated a clear separation between SOD1^G37R^ mice and controls (Fig. 4B). A volcano plot prominently highlighted upregulated genes related to immune cell activation (e.g., IL-2rg, CD44), complement system (e.g., C1qa, C1qc, C4b), and leukocyte migration (e.g., ICAM-1) (Additional file 1: Fig. S3A). Accordingly, Gene Ontology (GO) analysis highlights various processes related to immune system activation and inflammation (Fig. 4C and Additional file 6: Table S5) including an upregulation of genes related to immune system process (gene ratio: 0.2438, Padj = 8.278E−63) and cytokine production (gene ratio: 0.1003, Padj = 3.770E−38) (Fig. 4C, D). Furthermore, there was upregulation of genes associated with CD4 T-cell activation pathway (gene ratio: 0.0144, Padj = 9.505E−07); for instance, CD44 which contributes to the activation, migration, and effector functions of T cells; CD81 which plays a role in various aspects of CD4 T-cell activation and function, CD3 signaling, immune synapse formation and migration [43]; IL2RG which is a critical component of IL-2 receptor complexes, and its presence is essential for various CD4 T-cell functions, including activation, proliferation, differentiation, and immune regulation (Fig. 4C, D; Additional file 6: Table S5).

KEGG pathway analysis provides further insight into mechanisms that support CD4 T-cell migration and activity. These include upregulation of the leukocyte transendothelial migration pathway (gene ratio: 0.0417, Padj = 7.332E−11) [45], NF-kappa B signaling pathway (gene ratio: 0.0359 Padj = 6.816E−08), Th17 cell differentiation (gene ratio: 0.0281, Padj = 9.489E−05), and antigen processing and presentation (gene ratio: 0.0204, Padj = 0.00126) (Fig. 4E; Additional file 7: Table S6) [46, 47]. As indicated in GO analysis (Additional file 1: Fig. S3B), Fig. 4F depicts a detailed representation of the antigen processing and presentation pathway, emphasizing genes central to enzymatic processing of antigens in the endosome (i.e., AEP and CTSB) and genes related to MHCII complex assembly (i.e., Ii, MHCII and CTSB/L/S), which were notably enhanced in SOD1^G37R^ mice (red boxes). Together, these results strongly suggest that CD4 T cells play a crucial role in the neuroinflammation observed in SOD1^G37R^ mice, likely through their increased activation, altered trafficking, and potential skewing towards a more inflammatory phenotype.

### Infiltrating CD4 T cells interact with antigen presenting cells in spinal cords of mutant SOD1^G37R^ mice

**Fig. 5:**
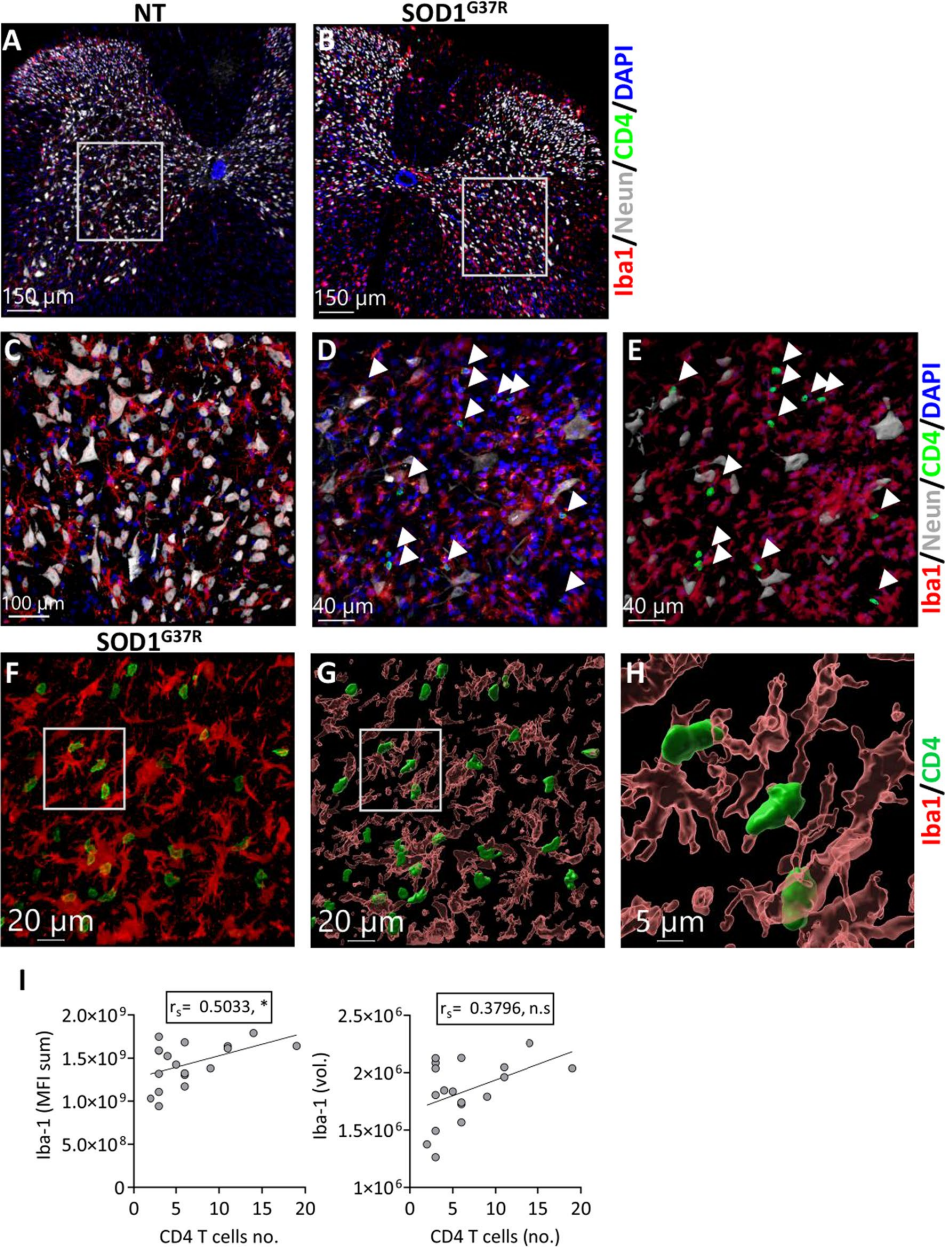

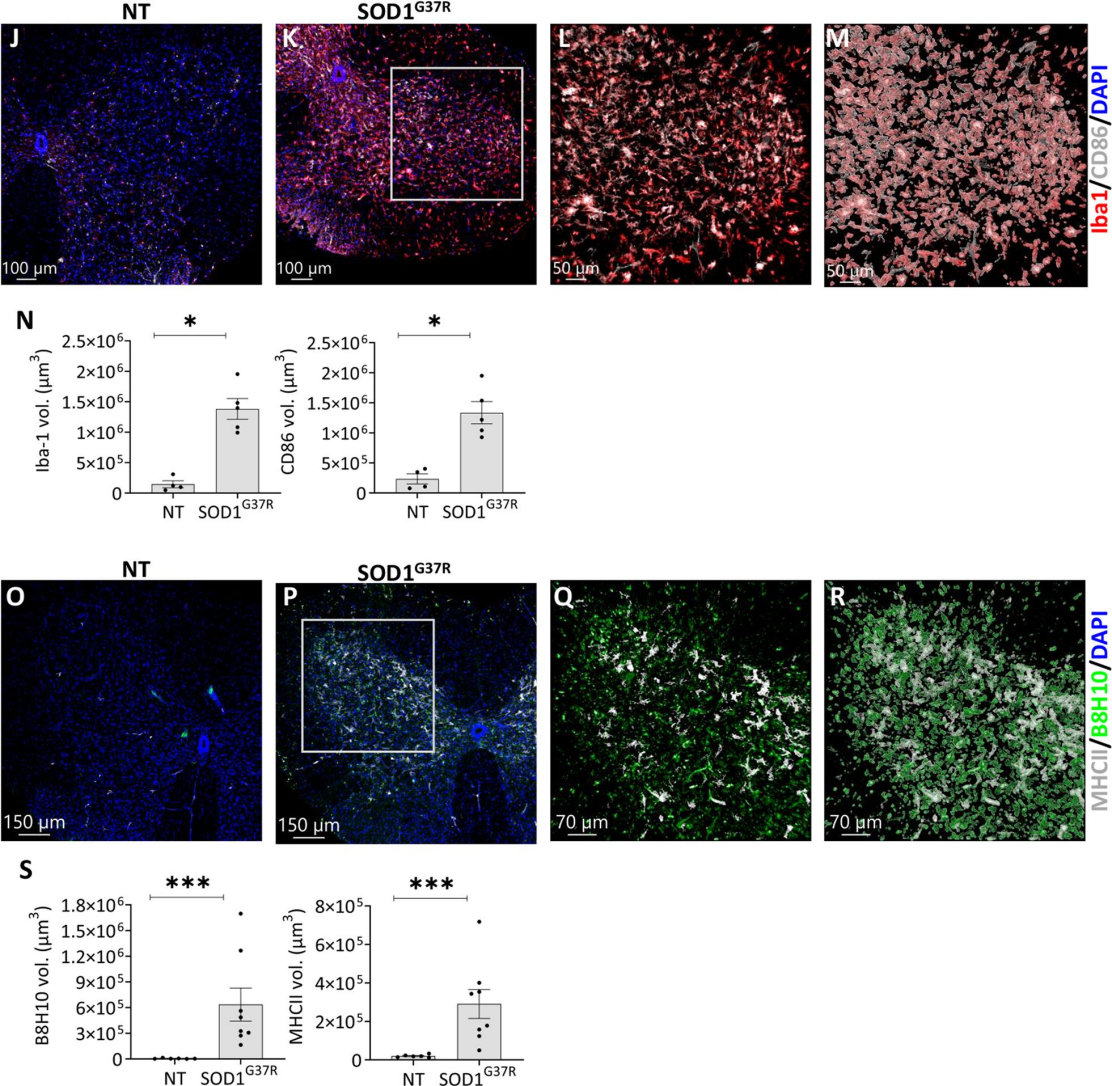
Infiltrating CD4 T cells interact with antigen presenting cells in spinal cords of mutant SOD1^G37R^ mice. Mice underwent euthanasia followed by perfusion with cold PBS. Thereafter, SCs were harvested, fixed, and transversely sectioned. For 3D visualization, a z-stack encompassing a minimum of 40 μm thickness was captured using a confocal microscope, with images acquired every 1 μm. **A**–**D** Lumbar SC sections of NT (**A**, **C**) and SOD1^G37R^ (**B**, **D**) mice, immunolabeled with anti-Iba1 (red), anti-NeuN (white), anti-CD4 (green), and DAPI (blue) (*n* = 3). Scale bars represent ×10 (**A**, **B**) and ×40 magnification, Z-stack (**C**, **D**). **E** 3D projection of z-sections from panel **D** using Imaris (Methods). **F** Lumbar SC section of symptomatic mutant SOD1^G37R^ mice (×60 magnification, Z-stack), immunolabeled with anti-Iba1 (red) and anti-CD4 (green) (*n* = 3). **G**, **H** 3D reconstruction of z-sections (from **F**) using Imaris. **I** Correlation between Iba-1 fluorescence intensity (left) or Iba-1 fluorescence volume (right) and frequencies of colocalized CD4 T cells (*n* = 6, 3 sections from different regions within the lumbar SC). *P ≤ 0.05, Spearman's rank correlation. **J**–**L** Lumbar SC sections of NT (*n* = 4) (**J**) and symptomatic mutant SOD1^G37R^ mice (*n* = 5) (**K**, **L**), immunolabeled with anti-Iba1 (red), anti-CD86 (white), and DAPI (blue) [×10 (**J**, **K**) and ×20 (**L**) magnification, z-stack). **M** 3D reconstruction of z-sections (from **L**) using Imaris. **N** Quantification of Iba-1 (left) and CD86 (right) fluorescence volume in SC sections from NT (*n* = 4) and mutant SOD1^G37R^ (*n* = 5) mice. *P ≤ 0.05, Mann–Whitney U test. **O**–**Q** Lumbar SC sections from NT (*n* = 6) (**O**) and symptomatic mutant SOD1^G37R^ mice (*n* = 8) **(P**, **Q)** [×10 magnification (**O**, **P**) and ×20 magnification, z-stack (**Q**)] immunolabeled with anti-MHCII (white), anti-B8H10 (green) and DAPI (blue). **R** 3D reconstruction of z-sections (from **Q**) using Imaris. **S** Quantification of misfolded aggregated SOD1 (left, B8H10) and MHCII (right) fluorescence volume in NT and mutant SOD1^G37R^. ***P ≤ 0.001, Mann–Whitney U test

To investigate the potential activation of effector CD4 T cells by microglia, we performed immunostaining on lumbar SC sections from SOD1^G37R^ mice, targeting both CD4 T cells and the microglial marker, Iba1. In line with our flow cytometry analysis (Fig. 3C), confocal image analysis revealed increased numbers of CD4 T cells in SC sections from SOD1^G37R^ mice as compared with age-matched littermate controls (Fig. 5A–E). In addition, CD4 T cells were in a close association with Iba1+ microglia (Fig. 5F–H). Notably, Iba1 MFI was found to be positively correlated with the number of infiltrating CD4 T cells. However, when considering the volume of Iba1-positive areas, there was no significant correlation with the number of infiltrating CD4 T cells (Fig. 5I). Concomitantly, there was an increase in the volume of Iba-1+ and CD86+ cells (Fig. 5J–N and Additional file 1: Fig. S4A, B), along with increased volume of MHCII+ cells in regions of aggregated misfolded SOD1 (Fig. 5O–S and Additional file 1: Fig. S4C, D). Collectively, our data suggest that active immunological synapse occurs between microglia and infiltrating effector CD4 T cells potentially modulating the neuroinflammatory process in SOD1^G37R^ mice.

## Discussion

In the present study, we sought to elucidate the contribution of T cells in the neuroinflammatory process of ALS, primarily using the late-onset SOD1 mouse model. First, we observed a skew towards effector memory phenotypes, which correlated with disease progression. Within the SC of SOD1^G37R^ mice, CD4 T cells showed primarily a proinflammatory phenotype which coincided with activated microglia and positively correlated with infiltrating CD8 T cells. RNA-seq analyses reinforced these observations, uncovering a robust adaptive immune response underscored by upregulated pathways related to CD4 T-cell activity and antigen presentation. These findings thus highlight an important role for age-related T-cell subsets in ALS progression.

In our work, we examined the immune compartment of mice at symptomatic disease stage, characterized by specific phenotypic markers such as foot-dragging or toe-curling during walking [42]. Specifically, SOD1^G37R^ mice were approximately 370 days of age and SOD1^G93A^ mice were approximately 130 days of age at the time of sample collection. This approach was intended to capture a representative similar state of disease progression in these models. Using the late-onset SOD1^G37R^ mice, we identified a pronounced representation of effector memory phenotypes, which suggest a heightened state of T-cell effector functions. This is in contrast to the dominance of naive T cells in the early-onset and commonly used SOD1^G93A^ mice, reflecting a landscape of reduced antigenic exposure along with increased turnover of naïve T cells [48–51]. These results are in line with the most noticeable changes observed in the overall T cell population with age; the reduced frequencies of naive T cells along with increased frequencies of exhausted and effector phenotypes [31, 48, 49] result in disrupted immune tolerance and consequently improper T cell reactions, excessive cytokine production, cytotoxic responses, and tissue damage [52, 53]. Such impact of age-related dysregulated T-cell effector functions were recently observed in patients with ALS [29] and several neurodegenerative processes such as MS [54], PD [55], and AD [56]. Together, our results suggest that late-onset mouse models more accurately reflect the complexity of immune responses occurring in ALS.

Neuroinflammation, particularly related to the activity and distribution of different T-cell subsets, is becoming increasingly recognized as a hallmark of ALS [20]. Research conducted on leukocytes and SCs from ALS patients and SOD1 mouse models has demonstrated a significant inflammatory component in disease pathobiology; peripheral blood cell counts of natural killer cells, granulocytes, CD3+, CD4+, CD8+ and CD3+CD56+T cells were all shown to increase in patients with ALS [28]. Furthermore, within the realm of CD4 T cells, a correlation between the proinflammatory Th1/Th17 phenotype and disease severity was documented [57] and Tregs were shown to be dysfunctional in both ALS patients [58] and SOD1^G93A^ mice [59]. Similarly, an augmented presence of expanding CD4 T cells was shown in CSF samples of ALS patients, which express lineage-defining transcription factors typical to CD4 CTLs, Th1, and Th2 cells [29]. Our results in the late-onset SOD1^G37R^ mice show a simultaneous rise in pro- and anti-inflammatory populations (Th1 and Th2) and a decrease in the CD4 CTL population. Furthermore, EM CD4 T cells and CD4 CTL populations correlated with weight loss in SOD1^G37R^ mice and thus highlight a possible association with disease progression. This pattern hints at a chronic inflammatory response which, along with the accumulation of dysregulated T cells, exacerbate the disease process [29, 57, 60]. However, in contrast to a previous study showing a correlation between IL-17+ cells and disease progression[57], our study neither observed significant changes in the frequency of Th17 cells—immunolabeled with anti-RORγt—nor their correlation with disease severity. This may be attributed either to the use of RORγt staining for the identification of Th17 cells or to the variation in disease stage or mechanism. In any case, the effector function of CNS infiltrating proinflammatory T cells and particularly Th17 cells may be extremely pathogenic in ALS and should be further explored. In addition, we found that while the frequency of circulating CD4 CTLs decreased, they correlated with disease progression, suggesting that they may accumulate within the CNS as part of the disease process [29]. Together, these results highlight the sophisticated, multifaceted immune dynamics in ALS, underscoring the need for further research to elucidate these intricate immune mechanisms and their implications for ALS therapy.

The intricate relationship between T cell activation, their infiltration into CNS borders and parenchyma, and the subsequent neuroinflammatory process are central to unveil pathological mechanisms underpinning neurodegenerative diseases [29, 61–64]. Several studies have indicated increased T-cell frequencies within the CNS of individuals with ALS [26, 65], and mouse models [66], with both CD4+ and CD8+ T cells observed adjacent to deteriorating neurons [26]. Here, we observed a significant infiltration of T cells into the SCs of SOD1^G37R^ mice, which was not observed to the same extent in SOD1^G93A^ mice. This aligns with previous findings showing minimal neuroinflammatory response in early clinical stages of ALS in SOD1^G93A^ mice [66]. Intriguingly, T cells from CSF of ALS patients were found to undergo clonal expansion, hinting at antigen-triggered activation within the CNS [67, 68]. In line with findings showing an increase in proinflammatory Th1 cells in the CSF of ALS [29] and MS patients [69], we showed that the majority of CD4 T cells in the SC of SOD1^G37R^ mice exhibited a Th1 phenotype and expressed the activation markers CD69, CD81 and the checkpoint molecule PD1 as part of their effector function within the CNS. Although activated CD8 T cells expressed both PD1 and Lag3**,** indicating a more exhausted phenotype [70–72], the overall T-cell response in the SC of SOD1^G37R^ mice appears extremely neurotoxic, yet to be fully explored [73–75].

APCs at the CNS barriers and/or parenchyma are essential for locally executing and regulating T-cell responses via MHC–TCR interactions [22, 25]. Our RNA-seq studies demonstrate upregulated genes related to CD4 T-cell activation (e.g., CD44, CD81 and Il2rg) along with upregulated genes related to antigen processing and presentation (e.g., MHCII complex related genes: H2-Aa, H2-Ab1, H2-Eb1, H2-DMa, H2-DMb). Recent studies have shown that within the CNS parenchyma, certain disease-associated microglial subsets exhibit APC phenotypes [45, 76] which either promote [64] or limit [77] pathology. In a rat model of PD, infiltration of CD4 and CD8 T cells into the brain was followed by increased frequencies of MHCII-expressing microglia along with enhanced neuronal loss [22]. Similar to ALS mice, RNA-seq analysis of immune cells in the brain of PD mice revealed enrichment in GO terms related to antigen processing and presentation and T-cell activation [78]. Targeting the expression of MHCII, resulted in reduced T-cell and monocyte infiltration into the brain along with attenuated neurodegeneration [79]. Together, these results suggest that T-cell activation within the CNS might be a central mechanism underlying the progression of neurodegenerative diseases such as PD and ALS [80].

**Fig. 6:**
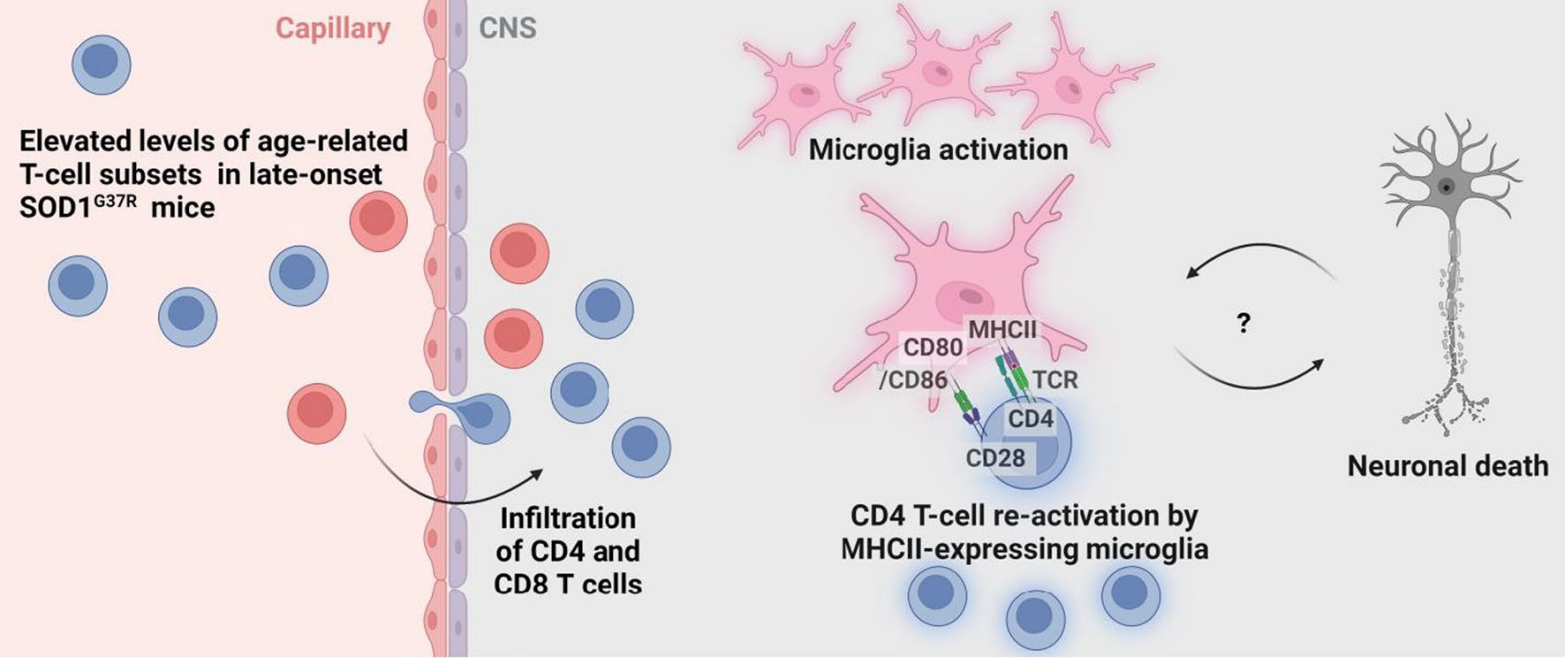
CD4 T-cell-mediated neuroinflammation in a late-onset mouse model of ALS. Elevated frequencies of systemic age-related T-cell subsets lead to neurotoxic inflammation in the lumbar SC, a process characterized by APCs activation, infiltration of CD4 (blue) and CD8 (red) T cells, and an upsurge in the expression of genes and proteins involved in antigen presentation and T cell activation. This cascade can be both a precursor to and a consequence of neuronal death

Providing a cellular context in SOD1^G37R^ mice, we show that CD4 T cells were in close proximity to microglia cells along with marked elevated expression of Iba1 and MHCII. Likewise, the association of Iba-1 levels with increased frequencies of infiltrating CD4 T cells, suggests a plausible causal relationship between T-cell infiltration and neurotoxic microglia activation in the course of ALS. Figure 6 depicts a possible scenario whereby CD4 T cells in spleens of late-onset SOD1^G37R^ show an age-related proinflammatory phenotype. Upon infiltration into the SC, these CD4 T cells are re-activated by antigen-presenting microglia cells, thereby facilitating their activation and furthering the inflammatory response, which could trigger release of neurotoxic factors leading to neuronal death. Our findings hint at a possible feedback loop, wherein the activation and interaction of CD4 T cells with microglia could lead to sustained neuroinflammation, possibly exacerbating disease progression [62].

Although the SOD1 mouse models exhibit similar neuropathology and neuroinflammation characteristics of ALS [18], the disease in human is substantially more heterogeneous due to genetic and environmental variations [81, 82]. Nonetheless, while the human disease is primarily age-related with the average age of patients around 60–70 years [2], the immunological changes occurring with age were mostly neglected in animal models. Moreover, despite the fact that these animal models remain essential for the development of optimized therapeutic drugs, in some cases, only human studies will definitely determine the real therapeutic value of these strategies. Thus, further human studies considering our findings can unveil various age-related T-cell subsets such as the increased frequencies of effector T cells and CD4 CTLs, which were also evident in the CSF of ALS patients, that can be promising targets to modify the disease process. The molecular profiles, signaling pathways, antigen specificities, and infiltration routes of such neurotoxic T cell subsets can all be valuable diagnostic and therapeutic targets. In addition, further understanding of the infiltration process and synapse formation of T cells with brain APCs can be harnessed to develop novel immunoregulatory approaches such as the use of antigen-specific Tregs or chimeric antigen receptor (CAR) T cells that target specific antigens within the CNS [83].

## Conclusions

To conclude, our investigations delineate the intricate immune dynamics in the context of both early and late-onset SOD1 mouse models of ALS, emphasizing a potential pathogenic role of infiltrating T cells. The contrasting predominance of effector memory phenotype in late-onset SOD1^G37R^ mice compared to naive T cells in early-onset SOD1^G93A^ suggests distinct immunological trajectories and, potentially, differing disease progression pathways. Our RNA-seq data underscore an amplified adaptive immune response within the SC of SOD1^G37R^ mice, hallmarked by an elevation in pathways related to CD4 T-cell activation and antigen presentation. This is further supported by our observations of CD4 T cells closely interacting with microglial cells, suggestive of potential antigen presentation and subsequent T cell activation. Together, these interactions hint at a self-sustaining feedback loop between CD4 T cells and microglia, driving persistent neuroinflammation and potentially accelerating ALS progression. Consequently, our insights extend the current understanding of immune involvement in ALS and offer valuable perspectives for potential therapeutic interventions targeting the immune dynamics in neurodegenerative diseases.

### Supplementary Information

**Additional file 1: Figure S1.** Flow cytometry gating strategy and immune alterations in spleens of SOD1 mice. **(A)** Gating strategy for spleen flow cytometry experiment for SOD1^G37R^ and SOD1^G93A^ mice. CD4 T-cell T populations were gated from lymphocytes (SSC-A vs FSC-A), live cells (Fixable Viability Dye vs FSC-A), and single cells (SSC-A vs SSC-H). **(B)** tSNE plot of CD4 T-cell population distribution of NT littermates of late-onset SOD1^G37R^ (*n* = 3) and early-onset SOD1^G93A^ mice (*n* = 3). **(C–N)** CD4 T-cell subsets frequency (mean ± SEM) of NT (*n* = 5) and symptomatic SOD1^G93A^ mice (*n* = 5), shown as percentage. **Figure S2:** CD4 and CD8 T-cell infiltration into the spinal cord of SOD1^G93A^ mice. **(A)** Gating strategy for SC flow cytometry experiment for SOD1^G37R^ and SOD1^G93A^ mice. **(B, C)** Event number of CD4 **(B)** and CD8 **(C)** T cells in SCs of NT mice (*n* = 7) and symptomatic mutant SOD1^G93A^ mice (*n* = 8). **Figure S3:** Elevation of genes related to inflammation and antigen presentation in SOD1^G37R^ mice. **(A)** Volcano plot of upregulated and downregulated genes related to inflammatory pathway. **(B)** Heatmap of differentially expressed genes in the SC from symptomatic mutant SOD1^G37R^ (*n* = 10) and NT mice (*n* = 10) that are involved in antigen processing and presentation (GO:0019882). **Figure S4:** Microglial expression of CD86 and MHCII in SCs of non-transgenic control mice. **(A)** Lumbar SC sections of NT (*n* = 4) mice. Sections were immunolabeled with anti-Iba-1 (red), anti-CD86 (white) and DAPI (blue) (×20 magnification, z-stack). **(B)** 3D reconstruction of z-sections (from **A**) using Imaris. **(C)** Lumbar SC sections of NT (*n* = 6) mice. Sections were immunolabeled with anti-MHCII (white), anti-B8H10 (green) and DAPI (blue) (×20 magnification, z-stack). **(D)** 3D reconstruction of z-sections (from **C**) using Imaris.

**Additional file 2: Table S1.** Antibodies used in flow cytometry experiments for SC cells and splenocytes. This table lists the antibodies used in the flow cytometry analysis of SCs and splenocytes. It includes details on conjugation, target antigens, the cell types for which each antibody was used, and the suppliers. This detailed categorization facilitates understanding of the specific reagents used in the experimental process.

**Additional file 3: Table S2.** Antibodies used in IHC experiments. This table lists the antibodies used in the IHC analysis of SC sections. The table includes information on the target antigen, the dilution used, the source of the antibody, and the species origin.

**Additional file 4: Table S3.** Frequencies of CD4 T-cell populations in SOD1 and littermate control mice. Percentages of CD4 T-cell populations derived from spleens of SOD1^G93A^, SOD1^G37R^ and littermate control mice are displayed. Data for each genotype are presented in terms of Avg. and SEM.

**Additional file 5: Table S4.** Differentially expressed genes in lumbar spinal cord sections of SOD1^G37R^ and littermate control mice. Lumbar SC RNA-seq results of 10 SOD1^G37R^ mice and 10 littermate control mice. Genes are detailed with their corresponding ENSEMBL ID and gene symbols, log2 fold change values, and adjusted p-values. Fold change values > 1 suggest upregulation whereas values < − 1 downregulation. Data analysis and organization were executed utilizing the NeatSeq Flow DeSeq2 module.

**Additional file 6: Table S5.** Upregulated Immune-related GO biological processes in lumbar spinal cords of SOD1^G37R^ mice. The table outlines the GO biological processes found to be upregulated in lumbar SC samples, as ascertained through bulk RNA-seq analysis. Columns from left to right present the biological process name, gene ratio, and p.adjust.

**Additional file 7: Table S6.** Upregulated Immune-related KEGG pathways in lumbar spinal cords of SOD1^G37R^ mice. The table delineates the KEGG pathways found to be upregulated in lumbar SC samples, as determined by bulk RNA-seq analysis. Columns from left to right represent the pathway name, gene ratio, and p.adjust.

## Data Availability

The RNA-seq data have been deposited to the GEO repository with accession number GSE246397.

## Abbreviations

ALS: Amyotrophic lateral sclerosis
APC: Antigen presenting cell
CNS: Central nervous system
EM: Effector memory
CD4 CTLs: Cytotoxic CD4 T cells
IHC: Immunohistochemistry
MHC: Major histocompatibility complex
SC: Spinal cord
