# Supplementary figures and images for: CD4 T-cell aging exacerbates neuroinflammation in a late-onset mouse model of amyotrophic lateral sclerosis
Source: J Neuroinflammation. 2024 Jan 11;21:17. doi: 10.1186/s12974-023-03007-1 (PMC10782641; doi:10.1186/s12974-023-03007-1)

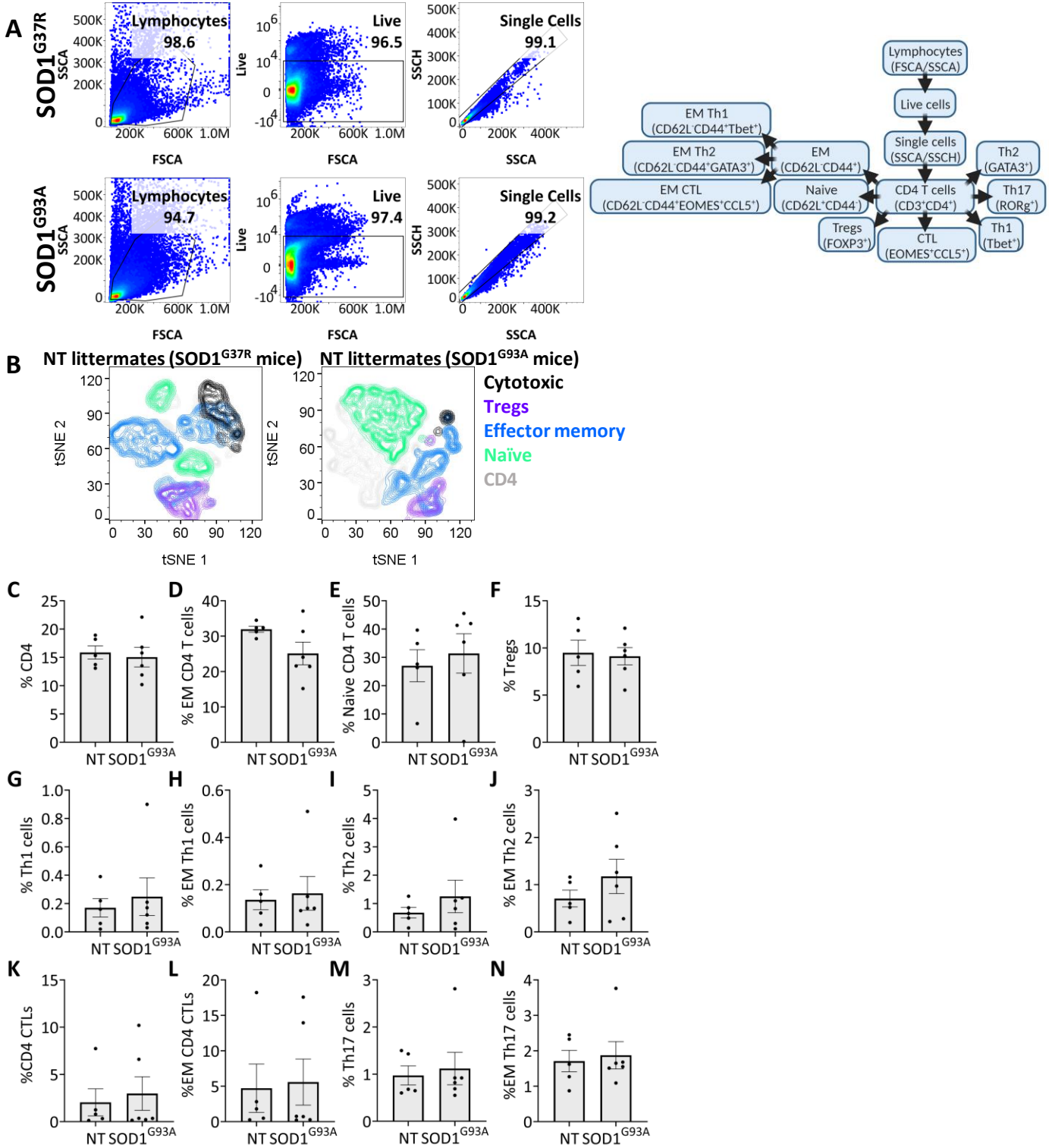

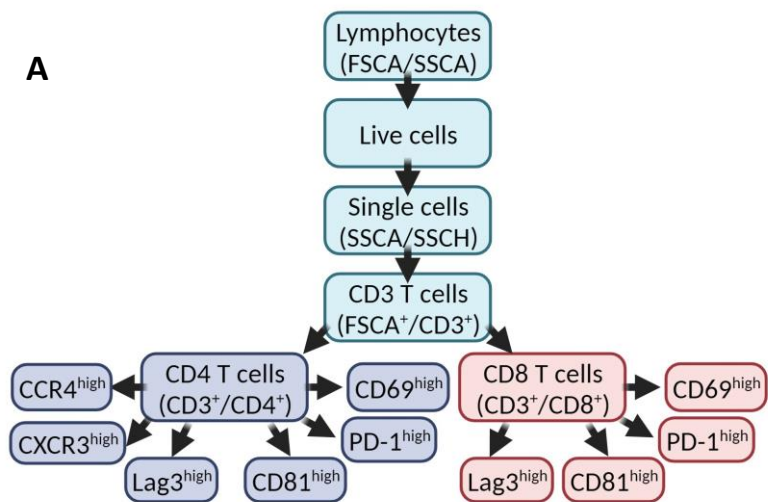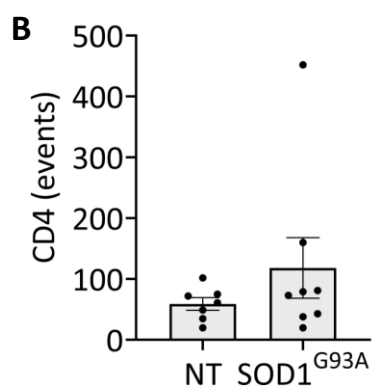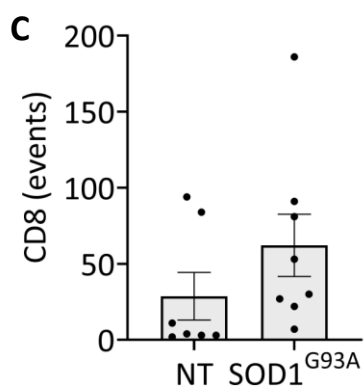

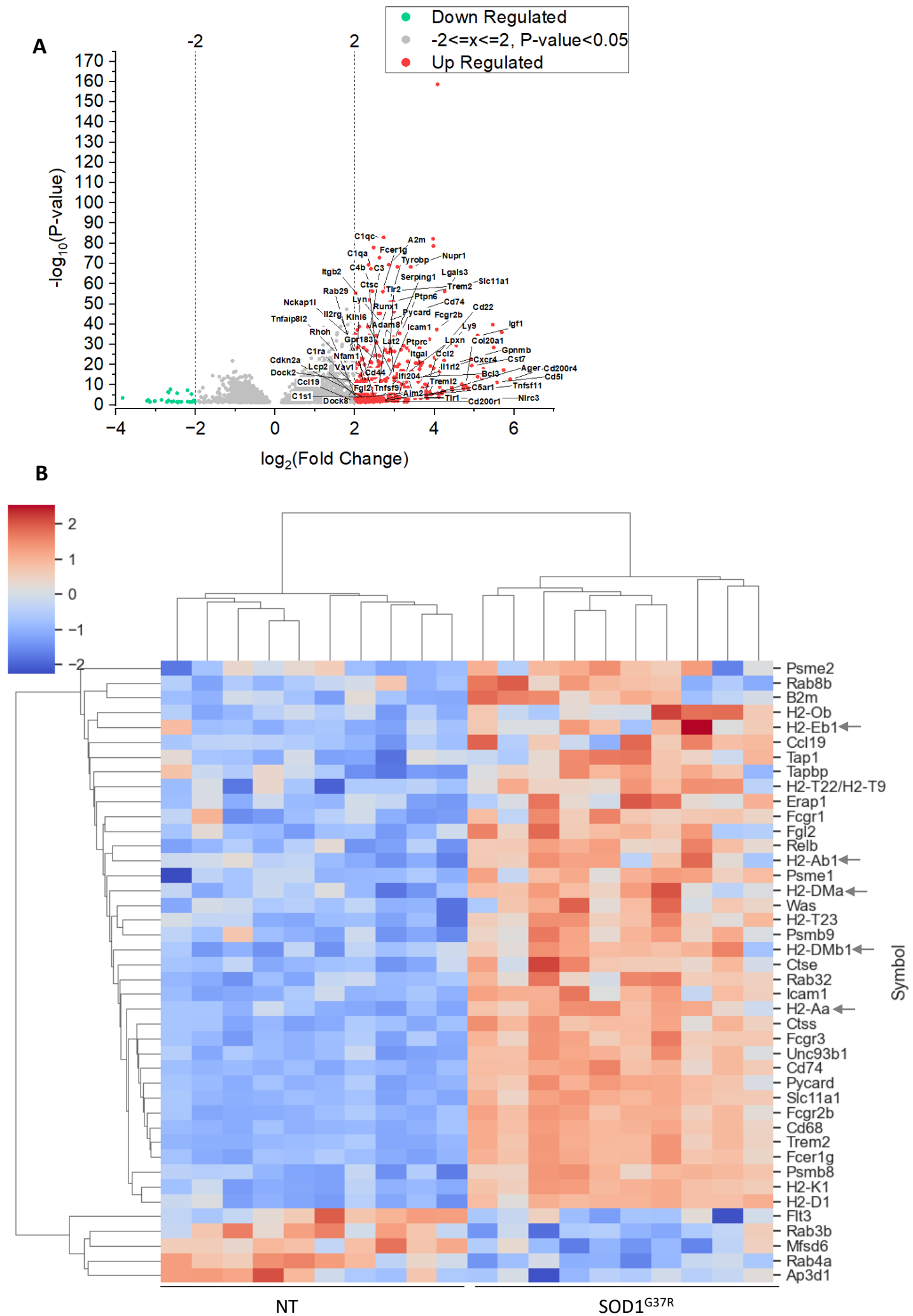

Supplementary Figure 3

NT

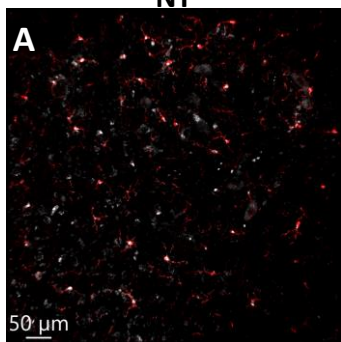

NT

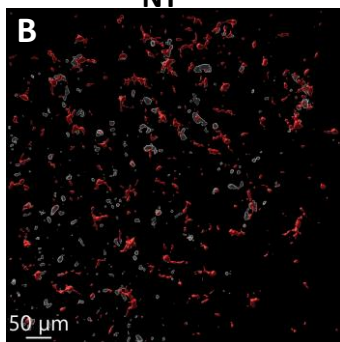

Iba1/CD86/DAPI

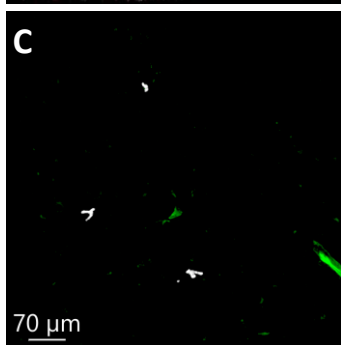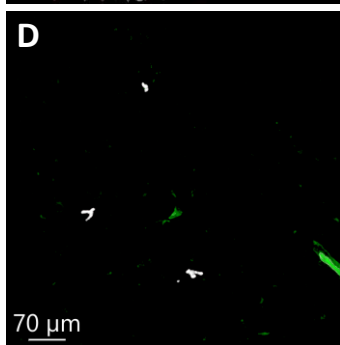

MHCII/B8H10/DAPI

Supplement: Supplementary file 1 — Additional file 1: Figure S1. Flow cytometry gating strategy and immune alterations in spleens of SOD1 mice. (A) Gating strategy for spleen flow cytometry experiment for SOD1G37R and SOD1G93A mice. CD4 T-cell T populations were gated from lymphocytes (SSC-A vs FSC-A), live cells (Fixable Viability Dye vs FSC-A), and single cells (SSC-A vs SSC-H). (B) tSNE plot of CD4 T-cell population distribution of NT littermates of late-onset SOD1G37R (n = 3) and early-onset SOD1G93A mice (n = 3). (C–N) CD4 T-cell subsets frequency (mean ± SEM) of NT (n = 5) and symptomatic SOD1G93A mice (n = 5), shown as percentage. Figure S2: CD4 and CD8 T-cell infiltration into the spinal cord of SOD1G93A mice. (A) Gating strategy for SC flow cytometry experiment for SOD1G37R and SOD1G93A mice. (B, C) Event number of CD4 (B) and CD8 (C) T cells in SCs of NT mice (n = 7) and symptomatic mutant SOD1G93A mice (n = 8). Figure S3: Elevation of genes related to inflammation and antigen presentation in SOD1G37R mice. (A) Volcano plot of upregulated and downregulated genes related to inflammatory pathway. (B) Heatmap of differentially expressed genes in the SC from symptomatic mutant SOD1G37R (n = 10) and NT mice (n = 10) that are involved in antigen processing and presentation (GO:0019882). Figure S4: Microglial expression of CD86 and MHCII in SCs of non-transgenic control mice. (A) Lumbar SC sections of NT (n = 4) mice. Sections were immunolabeled with anti-Iba-1 (red), anti-CD86 (white) and DAPI (blue) (×20 magnification, z-stack). (B) 3D reconstruction of z-sections (from A) using Imaris. (C) Lumbar SC sections of NT (n = 6) mice. Sections were immunolabeled with anti-MHCII (white), anti-B8H10 (green) and DAPI (blue) (×20 magnification, z-stack). (D) 3D reconstruction of z-sections (from C) using Imaris. [file 12974_2023_3007_MOESM1_ESM.pdf]
