# Supplementary material for: CD4 T-cell aging exacerbates neuroinflammation in a late-onset mouse model of amyotrophic lateral sclerosis
Source: J Neuroinflammation. 2024 Jan 11;21:17. doi: 10.1186/s12974-023-03007-1 (PMC10782641; doi:10.1186/s12974-023-03007-1)
Supplement: Supplementary file 2 — Additional file 2: Table S1. Antibodies used in flow cytometry experiments for SC cells and splenocytes. This table lists the antibodies used in the flow cytometry analysis of SCs and splenocytes. It includes details on conjugation, target antigens, the cell types for which each antibody was used, and the suppliers. This detailed categorization facilitates understanding of the specific reagents used in the experimental process. [file 12974_2023_3007_MOESM2_ESM.docx]

| Antibody Target | Conjugation | Supplier | Species | Application |
| --- | --- | --- | --- | --- |
| CD194 (CCR4) | Brilliant Violet 421 | Biolegend, CA, USA | Mouse | Extracellular Staining (SC cells) |
| CD4 | Brilliant Violet 510 | Biolegend, CA, USA | Mouse | Extracellular Staining (SC & Splenocytes) |
| CD279 (PD-1) | Brilliant Violet 605 | Biolegend, CA, USA | Mouse | Extracellular Staining (SC & Splenocytes) |
| CD223 (LAG-3) | Brilliant Violet 785 | Biolegend, CA, USA | Mouse | Extracellular Staining (SC cells) |
| CD69 | FITC | Thermo Fisher Scientific, MA, USA | Mouse | Extracellular Staining (SC cells) |
| CD8a | PerCP/Cyanine5.5 | Biolegend, CA, USA | Mouse | Extracellular Staining (SC cells) |
| CD183 (CXCR3) | PE | Thermo Fisher Scientific, MA, USA | Mouse | Extracellular Staining (SC cells) |
| CD196 (CCR6) | PE/Cyanine7 | Biolegend, CA, USA | Mouse | Extracellular Staining (SC cells) |
| CD81 | APC | Biolegend, CA, USA | Mouse | Extracellular Staining (SC cells) |
| CD3 | Alexa Fluor 700 | Thermo Fisher Scientific, MA, USA | Mouse | Extracellular Staining (SC cells & Splenocytes) |
| CD44 | Brilliant Violet 650 | Biolegend, CA, USA | Mouse | Extracellular Staining (Splenocytes) |
| CD62L (L-Selectin) | Alexa Fluor 700 | Thermo Fisher Scientific, MA, USA | Mouse | Extracellular Staining (Splenocytes) |
| RORγt | Brilliant Violet 421 | BD, NJ, USA | Mouse | Intracellular Staining (Splenocytes) |
| T-bet | Brilliant Violet 785 | Biolegend, CA, USA | Mouse | Intracellular Staining (Splenocytes) |
| FOXP3 | Alexa Fluor 488 | Biolegend, CA, USA | Mouse | Intracellular Staining (Splenocytes) |
| GATA3 | PerCP/Cyanine5.5 | Biolegend, CA, USA | Mouse | Intracellular Staining (Splenocytes) |
| CCL5 (RANTES) | PE | Biolegend, CA, USA | Mouse | Intracellular Staining (Splenocytes) |
| EOMES | PE-Cyanine7 | Thermo Fisher Scientific, MA, USA | Mouse | Intracellular Staining (Splenocytes) |
