# Supplementary material for: CD4 T-cell aging exacerbates neuroinflammation in a late-onset mouse model of amyotrophic lateral sclerosis
Source: J Neuroinflammation. 2024 Jan 11;21:17. doi: 10.1186/s12974-023-03007-1 (PMC10782641; doi:10.1186/s12974-023-03007-1)
Supplement: Supplementary file 3 — Additional file 3: Table S2. Antibodies used in IHC experiments. This table lists the antibodies used in the IHC analysis of SC sections. The table includes information on the target antigen, the dilution used, the source of the antibody, and the species origin. [file 12974_2023_3007_MOESM3_ESM.docx]

| Antibody target/ Fluorophore | Dilution | Supplier | Species | Application |
| --- | --- | --- | --- | --- |
| CD4 | 1:100 | Biolegend, CA, USA | Rat | Primary antibody |
| Iba-1 | 1:1000 | Abcam, United Kingdom | Goat | Primary antibody |
| Neun | 1:400 | Millipore, MA, USA | Mouse | Primary antibody |
| I-A/I-E (MHCII) | 1:100 | Biolegend, CA, USA | Rat | Primary antibody |
| B8H10 | 1:100 | MediMabs, QC, Canada | Mouse | Primary antibody |
| CD86 | 1:100 | BD, NJ, USA | Rat | Primary antibody |
| Alexa Fluor 488 | 1:500-250 | Invitrogen, MA, USA | Donkey | Secondary antibody |
| Alexa Fluor 546 | 1:500-250 | Invitrogen, MA, USA | Donkey | Secondary antibody |
| Alexa Fluor 633 | 1:250 | Invitrogen, MA, USA | Donkey | Secondary antibody |
| DAPI | 1:3000 | Biolegend, CA, USA | - | Nuclear staining |
