# Supplementary material for: CD4 T-cell aging exacerbates neuroinflammation in a late-onset mouse model of amyotrophic lateral sclerosis
Source: J Neuroinflammation. 2024 Jan 11;21:17. doi: 10.1186/s12974-023-03007-1 (PMC10782641; doi:10.1186/s12974-023-03007-1)
Supplement: Supplementary file 4 — Additional file 4: Table S3. Frequencies of CD4 T-cell populations in SOD1 and littermate control mice. Percentages of CD4 T-cell populations derived from spleens of SOD1G93A, SOD1G37R and littermate control mice are displayed. Data for each genotype are presented in terms of Avg. and SEM. [file 12974_2023_3007_MOESM4_ESM.docx]

|  |  | %CD4 | %EM CD4 T cells | % Naïve CD4 T cells | %Tregs | %Th17 cells | %EM Th17 cells | %Th1 cells | %EM Th1 cells | % Th2 cells | %EM Th2 cells | %CD4 CTLs | %EM CD4 CTLs |
| --- | --- | --- | --- | --- | --- | --- | --- | --- | --- | --- | --- | --- | --- |
| NT (SOD1G37R) | Avg. | 14.035 | 59.075 | 18.832 | 15.081 | 0.614 | 0.729 | 0.165 | 0.1 | 0.705 | 0.574 | 15.346 | 21.504 |
|  | SEM | 0.885 | 3.637 | 2.42 | 2.151 | 0.088 | 0.1 | 0.048 | 0.024 | 0.116 | 0.091 | 2.202 | 3.9 |
| SOD1G37R | Avg. | 14.979 | 61.826 | 21.032 | 17.479 | 0.533 | 0.681 | 0.343 | 0.183 | 1.067 | 0.702 | 8.608 | 12.128 |
|  | SEM | 0.628 | 2.491 | 1.955 | 0.753 | 0.042 | 0.056 | 0.057 | 0.032 | 0.119 | 0.066 | 1.02 | 1.512 |
| NT (SOD1G93A) | Avg. | 15.858 | 31.954 | 27.034 | 9.49 | 0.972 | 1.708 | 0.17 | 0.136 | 0.678 | 0.706 | 2.046 | 4.716 |
|  | SEM | 1.169 | 0.85 | 5.651 | 1.334 | 0.202 | 0.303 | 0.065 | 0.042 | 0.186 | 0.177 | 1.434 | 3.405 |
| SOD1G93A | Avg. | 15.047 | 25.108 | 31.395 | 9.118 | 1.118 | 1.873 | 0.248 | 0.163 | 1.252 | 1.175 | 2.978 | 5.582 |
|  | SEM | 1.725 | 3.206 | 6.959 | 0.92 | 0.343 | 0.387 | 0.133 | 0.071 | 0.573 | 0.363 | 1.775 | 3.254 |
